# Flipped-classroom training in advanced cardiopulmonary life support

**DOI:** 10.1371/journal.pone.0203114

**Published:** 2018-09-05

**Authors:** Jin Ho Beom, Ji Hoon Kim, Hyun Soo Chung, Su Mi Kim, Dong Ryul Ko, Junho Cho

**Affiliations:** 1 Department of Emergency Medicine, Yonsei University College of Medicine, Seoul, Korea; 2 Clinical Simulation Center, Yonsei University College of Medicine, Seoul, Korea; Nantes University Hospital, FRANCE

## Abstract

**Background:**

The effects of the flipped classroom have been demonstrated in various fields of education in recent years. Training in emergency medicine is also beginning to gradually implement the flipped classroom; however, its practical effect in emergency medicine contexts is not yet clear.

**Objective:**

The present study investigates the effects of the flipped classroom on advanced cardiopulmonary life support (ACLS) training implemented among practicum students in emergency medicine.

**Methods:**

The study randomly assigned into control and experimental conditions 108 fourth year students in the College of Medicine at Yonsei University, in Seoul, who were scheduled to take clinical practice in emergency medicine between March and July 2017. Students were taught about ACLS in either a traditional lecture-based classroom (control condition) or a flipped classroom (experimental condition); then, simulation training with ACLS scenarios was carried out. Finally, each student was rated on performance using a rating form developed in advance.

**Results:**

ACLS simulation scores of the students in the flipped classroom were 70.9±10.9, which was higher than those of the students in the traditional classroom (67.1±11.3); however, this difference was not statistically significant (p = 0.339). In addition, the difference in student satisfaction as measured on a survey was statistically insignificant (p = 0.655).

**Conclusions:**

Competency assessment after simulation-based training in ACLS undergone by senior medical students randomly assigned to flipped and traditional classrooms showed no statistical difference in competency between the two groups.

## Introduction

The concept of the “flipped classroom” was first implemented in 2012 [1]; since then, its effect has been demonstrated in various fields of education. Unlike in the traditional classroom, students in the flipped classroom review material and obtain knowledge ahead of class, and engage in group activities or projects relying on that knowledge in class [1–6]. Effects of flipped learning stemming from this element of active participation of students have been shown in various contexts. Most students have shown higher posttest scores compared with pretest scores and higher student satisfaction with the class when taught in a flipped classroom than in a traditional classroom [3,7–9]. It has also been reported, however, that national medical licensing examination scores showed no difference between types of instruction [6,7]. In one study, students scored higher on a skills test for reading an electrocardiogram in the flipped classroom, and spent more time studying alone before class than those who learned in a lecture-based classroom [10]. Belfi et al. compared pre- and posttest scores among students who underwent training in radiology in the flipped classroom, in the lecture-based classroom, and in self-study, respectively; although students in all three groups showed increase in scores after training, the greatest improvement was in the flipped-classroom students, while the other two groups improved at similar, lower levels [11].

The training paradigm of medical schools is shifting to one of performance-based training, because medical students need to develop competencies in medical care for successful performance of their future responsibilities, in addition to the medical knowledge that doctors must have [2,12]. Developing competencies requires accumulating hands-on experience treating actual patients and cannot be based on simple knowledge transfer, memorization, or written tests. In order to assess the competencies of graduating medical students, the Republic of Korea began implementing the Objective Structured Clinical Examination (OSCE) and Clinical Performance Examination (CPX) its National Medical Licensing Examination in 2010. The basis for incorporating these tests was the argument proposed in “Miller’s Pyramid” that the assessment of “knows” (i.e., assessing knowledge level using a written test) is the most primitive stage of assessment, and that assessment needs to move toward assessing “shows” (i.e., assessing whether the student can demonstrate and implement what he or she learned) [13].

The importance of competency in actual medical treatment is especially high in emergency response, such as response to cardiac arrest, because the seriousness of failure to perform properly under psychological and time pressures in emergency is not mitigated whatever the practitioner’s level of abstract knowledge. For this reason, training in emergency medicine is also beginning to gradually implement the flipped classroom; however, its practical effect in emergency medicine contexts is not yet clear. Lew reported that both students and teachers in emergency medicine rated the flipped classroom high in terms of satisfaction with the class, in a session teaching an approach to chest pain [14]. Tan et al. reported similar results for an emergency medicine practicum [15]. Rose et al. found that residents in emergency medicine who had learned pulmonary resuscitation of children through simulation and skills training scored higher on posttest than pretest using multiple-choice questions and on satisfaction [16].A study on teaching advanced cardiopulmonary life support (ACLS) found that students’ scores on various written tests improved when the flipped classroom was used [17]. These studies, however, did not assess students’ actual competency.

It is important for ACLS to be taught using a performance-based approach and evaluated with competency-based methods as opposed to written tests. However, teaching students the required knowledge and skills by actually allowing them to treat patients with cardiac arrest has many practical and ethical complications. Therefore, the present study investigates the effects of the flipped classroom as a means of teaching ACLS to clinical clerkship students in emergency medicine using a simulation approach.

## Methods

### Study design and participants

This study was randomized and single blinded. We randomly assigned to experimental and control groups 108 fourth-year students in the College of Medicine at Yonsei University, in South Korea, who were scheduled to be in clinical practice in emergency medicine between March and July 2017. The control group learned ACLS in a traditional lecture-based classroom, and the experimental group in a flipped classroom. We assigned students randomly to groups using computer software in February; of 108 students, 53 were assigned to the traditional lecture-based classroom and 55 to the flipped classroom.

During the student orientation on the first day of clinical practice for each group, one of the researchers briefed students on the study, handed out the study participation consent forms, asked them to read the form, had a Q&A session with the students regarding the study, and asked for their consent to participate. This researcher was in a position uninvolved in student grades and evaluations. The researcher informed students that their participation would have no bearing on their course grades and that their participation status would not be processed until their course grades had been submitted. Students had at least eight hours to make their decision on participation, and submitted the consent form in a sealed envelope at the end of the practicum period. We received the submitted consent forms after students’ final course grades in practical training had been submitted to the College of Medicine, that is, upon completion of practical training for both groups. The faculty members who evaluated the educational effects of the two teaching methods using the simulation were unaware of the status of individual students regarding study participation or of the assigned teaching method. Students who insisted on a particular teaching method or who did not attend the ACLS training classes or the simulation assessment session were excluded from the study.

The primary outcome was simulation rating scores and the secondary outcome was the students’ satisfaction survey.

This study was approved by Institutional Review Board of Severance Hospital [Approval No. 4-2016-0415] and the Clinical Research Information Service (CRIS); Korea Centers for Disease Control and Prevention, Ministry of Health and Welfare (Republic of Korea) [KCT0002416].

### Study protocol

Practical training in emergency medicine lasted for two weeks, including a one-hour class on ACLS and two hours of simulation. In the traditional classroom, the instructor gave a one-hour lecture using PowerPoint slides and held a Q&A session. In the flipped classroom, the instructor emailed students a PowerPoint file with a recorded explanation three days before the class, to allow them to study in advance; then, in class, students watched a video of an example of ACLS and engaged in group discussion. The video contained a “bad case” scenario developed by the Korean Association of Cardiopulmonary Resuscitation for educational purposes. After watching the video, students were asked to find and correct the errors in the treatment procedure, in teams (3 or 4 students). After the ACLS class, students received two hours’ simulation training to reduce potential difference in understanding of the simulation and underwent assessment of the educational effects in a familiar environment. In addition, to maintain objectivity, different faculty members separately conducted classroom instruction, simulation training, and student assessment, and faculty members ensured that all student groups received the same instructional content throughout the study period. On the last day of practical training, students in both groups performed an identical ACLS scenario on a simulation mannequin (Laerdal Medical, Stavanger, Norway), in teams of three or four; the researchers scored their performance as a team using a rating form developed in advance. Students were trained in the simulation in separate rooms with different access routes, to prevent exchange of information on the simulation. In addition, to determine whether any differences in assessment results of the two groups were due to features of that particular simulation, the OSCE and CPX scores that were scored during the emergency medicine clerkship were compared and they also went advanced trauma life support (ATLS) simulation training and assessment, conducted in the same manner as for ACLS.

The simulation rating form consisted of items on individual skills and a global rating. A total of 13 items on skills concerned whether each skill was performed properly; each item scored one point if the skill was demonstrated. The global rating item scored up to five points based on the assessment of overall performance on the simulation. (Table 1)

**Table 1:** Simulation rating form.

	Definition
Q1	Did the team use a defibrillator monitor?
Q2	Did the team check if the patient was conscious?
Q3	Pulse palpation and confirmation (a point given if the team checked the pulse through the carotid artery or femoral artery for at least 5 seconds)
Q4	Detecting cardiac arrest (a point given if the team made a statement of cardiac arrest or performed chest compression immediately)
Q5	Code blue activation (a point given if requesting help or a speaker announcement)
Q6	Checking the pulse rhythm for cardiac arrest (a point given if the team mentioned the pulse rhythm during Asystole, PEA, VF, and VT)
Q7	Performing defibrillation (in the cases of VF and VT, a point given if the team performed defibrillation and all of the following: - Keeping the energy level at a proper setting. - Ensuring no one including the performer himself or herself was in contact with the patient prior to pressing the discharge button. - Pressing the paddle on the patient’s chest with sufficient pressure.)
Q8	Performing chest compression after defibrillation (a point given only when the team performed chest compression immediately after defibrillation)
Q9	Proper administration of epinephrine (a point given when the team administered 1 mg epinephrine with 20 cc saline push by lifting the patient’s upper and lower limbs)
Q10	Checking self-circulation (a point given if the team checked the pulse through the carotid artery or femoral artery)
Q11	Securing correct airway (a point given for a successful intubation or use of i-gel)
Q12	A point is given for correct chest compression if the student does all of the following: - Placing a hand on a correct position (half below sternum). - Placing a hard board under the back of the patient. - Performing chest compression at a proper speed (100–120 times per minute). - Performing chest compression at a proper depth (5–6 cm). - Maintaining a proper shape of the hand and keeping the arm perpendicular to the ground with an open elbow during pressing. - Ensuring the chest fully relaxes after chest compression.
Q13	Determining the cause of cardiac arrest (a point given if the team has a discussion mentioning 5H and 5T)
Q Sum	Sum of items performed for Q1 through Q13
Q14	Global rating (between 1 and 5 points)

PEA: Pulseless Electric Activity, VF: Ventricular Fibrillation, VT: Ventricular Tachycardia

The highest possible score depended on the scenario: 18 points if fibrillation was necessary and 17 if not. We converted scores to a 100-point scale. Due to the nature of simulation training, scores applied to the whole team instead of each individual. After the assessment, the participants completed a survey to determine their satisfaction with the respective methods of instruction they had experienced by grading between 1 to 10 points by themselves (Table 2).

**Table 2:** Participant survey.

	****Survey Items (grade1–10 points)****
R1	It was generally a good class.
R2	I gained a lot of knowledge from this class.
R3	I actively participated in this class.
R4	The amount of time assigned to this class was adequate.
R5	The level of difficulty of this class was appropriate.
R6	I gained sufficient understanding of the content through this class.
R7	This class was interesting to me.
R8	I would recommend this class to other students.
R Sum	Sum of R1 through R8

### Statistical analysis

We calculated sample sizes for a two-sample means test using previous study data of 2 teams (181 students) with a traditional classroom group means of 8.03, SD±1.01, and a flipped classroom group means of 8.72, SD±1.01, in the ECG interpretation score [18]. Based on this data, the sample size was obtained using PASS (version 12, NCSS, LLC, Kaysville, Utah, USA). We estimated that a sample size of 53 pairs (106 participants) would be sufficient to evaluate the primary outcome at a significance level of 0.05 (two-sided) with 80% power, considering a 10% dropout rate.

We used R version 3.2.2 (The R Foundation for Statistical Computing, Vienna, Austria) for the statistical analyses. We performed score comparison using t-test and Wilcoxon rank-sum test, and frequency comparison using chi-squared test and Fisher’s exact test. For all tests, we defined p-value <0.05 as statistically significant.

## Results

**Fig 1:**
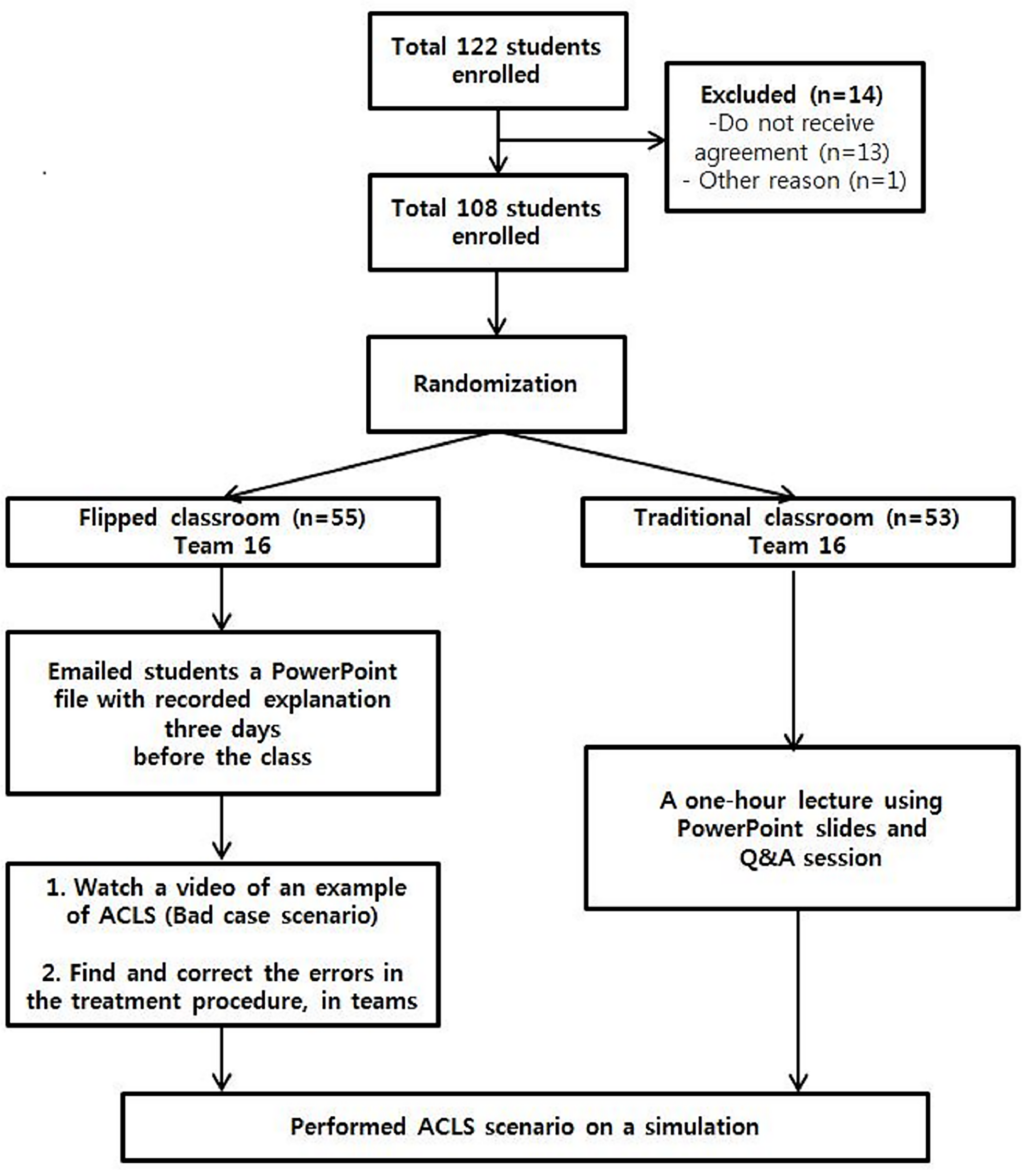
Flow diagram of participant eligibility and each group's teaching method. ACLS: Advanced Cardiopulmonary Life Support.

Out of 122 practicum students during the study period, 108 participated in the study. The 14 students who did not participate included 13 students who had already received training before preparation for the study was complete, and thus could not consent to participation, and one student who could not participate in clinical practice due to illness. (Fig 1)

We present demographic information on study participants in Table 3.

**Table 3:** Demographic characteristics and baseline of clinical performances.

Variable	Traditional Classroom (n = 53)	Flipped Classroom (n = 55)	p-value (<0.05)
Age (year)	26.4± 2.3	26.4 ± 2.2	0.46
Sex			0.15
Male	37(69.8)	31(56.3)	
Female	16(30.2)	24(43.6)	
Course of study			0.58
Medical graduate school	24(45.3)	22(40.0)	
Medical school	29(54.7)	33(60.0)	
ATLS	70.9±11.7	74.8 ±7.0	0.26
OSCE	90.0±6.6	85.0±9.9	0.10
CPX	64.7±5.0	65.9±5.8	0.52

Data are presented as mean ± standard deviation or *n* (%).

ATLS: Advanced Trauma Life Support, OSCE: Objective Structured Clinical Examination, CPX: Clinical Performance Examination.

ACLS simulation scores of the students in the flipped classroom were 70.9±10.9, which was higher than those of the students in the traditional classroom (67.1±11.3); however, the difference was not statistically significant (p = 0.339). The difference in student satisfaction as measured on the survey was also statistically insignificant (p = 0.655).

The students in the flipped classroom showed higher scores on the following individual items: checking if the patient is conscious, requesting help or speaker announcement, and properly administering epinephrine. The students in the lecture-based classroom showed higher scores only on checking at 2-minute intervals to see if self-circulation was restored (Table 4)

**Table 4:** ACLS simulation score and participant survey score.

Variable	Traditional Classroom (n = 53) Team 16	Flipped Classroom (n = 55) Team 16	p-value (<0.05)
Q1	16(100.0)	12(75.0)	0.1
Q2	9(56.3)	16(100.0)	0.007
Q3	14(87.5)	16(100.0)	0.484
Q4	15(93.7)	11(68.7)	0.172
Q5	0(0.0)	8(50.0)	0.002
Q6	12(75.0)	13(81.3)	>0.999
Q7	7(70.0)	8(100.0)	0.216
Q8	10(100.0)	7(100.0)	NA
Q9	6(37.5)	14(93.3)	0.001
Q10	16(100.0)	9(56.3)	0.007
Q11	13(81.3)	10(62.5)	0.433
Q12	5(31.3)	7(43.8)	0.716
Q13	4(25.0)	6(37.5)	0.446
Q sum (sum of items performed)	7.9±1.7	8.6±1.8	0.313
Q14 (global rating) between 1 and 5 points			0.767
1	0(0)	0(0)	
2	1(6.3)	1(6.3)	
3	6(37.5)	7(43.8)	
4	7(43.8)	8(50.0)	
5	2(12.5)	0(0.0)	
Total (Q sum + Q14)	11.6±2.1	12.0±2.2	0.568
ACLS (100-point conversion score)	67.1±11.3	70.9±10.9	0.339
Participant survey			
R1	8.6±1.6	8.4±1.0	0.7
R2	8.3±1.7	8.2±1.3	0.907
R3	7.7±2.0	8.31±0.9	0.259
R4	8.3±1.8	8.38±1.2	0.817
R5	8.7±1.1	8.12±1.5	0.233
R6	8.2±1.4	8.56±1.3	0.428
R7	8.1±1.8	8.69±1.3	0.264
R8	8.1±2.1	8.75±1.2	0.26
R sum (R1 through R8)	65.8±11.5	67.4±8.7	0.655

## Discussion

A variety of efforts are being made to shift the curriculum of medical training from a teacher-centered to a learner-centered model. This is because modern medical training is inefficient and inflexible, and focuses on getting good test scores instead of developing professional competencies [2,19]. The flipped classroom is an effective teaching method for changing students from passive to active learners and creating integrative educational strategies, as the teacher provides students with class materials ahead of time for students to study alone, while classroom activities such as discussion that require students’ active participation are the focus during the class [20].

The flipped classroom demonstrates better outcomes than the traditional lecture-based approach for students’ comprehension and application of course content [21]. A previous study compared the written test results of practicum students in emergency medicine who had learned about ACLS in a flipped classroom versus those who had learned in a regular classroom using lecture and simulation, and showed that the students in the flipped classroom scored higher than the other students [17]. The present study randomly assigned senior medical students on their practicum in emergency medicine to learn ACLS in two groups, one in a flipped classroom and one in a traditional lecture-based classroom. Competency assessment based on simulation performance results showed no statistically significant difference between groups. However, the observed difference was, although not statistically significant, tending toward 100 points which is important because our checklists were made through the essential process of ACLS. The success of ACLS does not merely depend on any one particular action or process. It depends on the results of the appropriation of the whole action or process.

There are other studies that do not demonstrate any effect of the flipped classroom. Chen et al. conducted a systematic review of nine studies that evaluated the flipped classroom in medical training, and reported that its effect on knowledge and skills change was inconclusive, as the statistical data required to estimate effect size could be obtained from only four studies [22]. A study with students in an emergency medicine practicum similarly found that those who had learned in a flipped classroom did not show more positive outcomes than those in a traditional classroom on a multiple-choice test; however, due to the lack of data on how well students had prepared for the class in the flipped classroom, this study’s findings may have been limited in value [23]. In another study, on the comparative effects of the flipped classroom for students on their ophthalmology practicum, final test scores showed no significant difference between treatment and control groups [24]. It has even been noted that, although the flipped classroom is an attractive teaching method for residents who do not have enough time for class, actual results show that only satisfaction with the method, not its substantive educational effect, is high [25]. Finally, a study of a neuroanatomy course showed that the flipped classroom evidenced no differences in test scores or in satisfaction from the traditional teaching method, which the investigator of the study interpreted as being because there was too much content to teach [26].

Overall, research that has demonstrated the efficacy of the flipped classroom has been mostly based on the comparison of written test results. Written tests are certainly an easy way to assess students; however, since medical training needs to develop and assess actual practical competencies for medical treatment, it remains important to conduct more studies assessing competencies, whether using simulations or in the actual job setting, instead of simply comparing written test results to evaluate the educational effects of the flipped-classroom method.

Our study has some limitations. First, where most previous studies in this area have focused on students’ satisfaction and changes in knowledge and skills, and have failed to compare student outcomes in higher domains, for example as presented in Kirkpatrick’s classification (e.g., change in behavior, professional competencies, patient outcomes), the present study focused on competencies, teamwork, and problem-solving skills using simulation as an assessment method; however, this study could not compare patient outcomes or long-term behavior change because of the limitations of the simulation and time limitations. Second, we could not find an objective way to measure students’ compliance. In a different context, Heitz et al. reported that only 31% of students failed to properly prepare for class [23]. In the present study, it is likely that the discussion format used in the flipped classroom placed pressure on students to prepare for the class; however, there are no relevant data. Third, we could not eliminate the possibility of the cross-contamination issue because students were not bound to their group assignment, and therefore could study with students from the other group. However, within the group, students were trained on simulation in separate rooms with different access routes, to ensure they did not share information on the simulation. Also, we did not reveal the checklists to students to ensure they did not find out what was going to happen before the simulation. Fourth, we have the limitation of being unable to be free of multiple testing issues while verifying each hypothesis for multiple questions.

Finally, due to the structure of the medical school practicum, we implemented training for only two weeks, even though there were many required items to teach; consequently, only one hour of the flipped classroom was applied to ACLS training. Thus, the minimum number of hours needed for the flipped classroom to be effective has not yet been established, and further study will be needed on these issues.

Nevertheless, it is worth appreciating the fact that almost all students and teachers preferred the flipped classroom, although the studies found only limited or no effects, because motivation is the first step for adult learners to gain educational benefits.

## Conclusions

An ACLS competency assessment conducted using a simulation after instruction in ACLS undergone by senior medical students randomly assigned to either a flipped classroom or a traditional lecture-based classroom showed no statistically significant difference between the two teaching methods.

**Appendix**

**ACLS Scenario: Pulmonary Embolism→Non-Shockable Arrest**

■ A 56-year-old male patient complained of difficulty breathing at dawn and visited the emergency room. The patient was diagnosed with lung cancer and malignant pleural effusion.
■ When he arrived in the emergency room, his mental state was drowsy (moaning).
■ Vital signs:
  Blood pressure 80/60 mmHg; pulse rate 68 times/min; respiration rate 24 times/min
  Body temperature 36.8 degrees Celsius; oxygen saturation 92%
  ECG normal sinus rhythm; lung sound clear
■ Adjust the oxygen saturation pattern according to the oxygen therapy
■ Patient’s electrocardiogram rhythm suddenly turns into pulseless electrical activity (PEA) and consciousness disappears.
■ Ensure that the treatment is conducted according to the non-shockable rhythm algorithm.
■ Debriefing points: non-shockable rhythm + PEA + reversible causes (5H & 5T)

**ACLS Scenario: Hyperkalemia→Shockable Arrest**

■ A 56-year-old male patient complained of swollen face and decreased urination in recent months. Today he complained of dizziness and fainted and visited the emergency room.
■ When he arrived in the emergency room, his mental state was drowsy (moaning).
■ Vital signs:
  Blood pressure 80/60 mmHg; pulse rate 110times/min; respiration rate 24 times/min;
  Body temperature 36.8 degrees Celsius; oxygen saturation 92%;
  ECG Tall T-wave, wide QRS
■ Adjust the oxygen saturation pattern according to the oxygen therapy
■ Patient's electrocardiogram rhythm suddenly turns into wide QRS (ventricular tachycardia) and ventricular fibrillation, and consciousness disappears.
■ Ensure that the treatment is conducted according to the shockable rhythm algorithm
■ the participant suspects hyperkalemia and confirms the lab results and administers Ca++ agent and NaHCO3, let spontaneous circulation return (ROSC)
■ If the participant continues cardiopulmonary resuscitation (CPR) without suspicion of hyperkalemia, K+ value is confirmed at appropriate time (11mEq/L)
■ Debriefing points: shockable rhythm + ventricular tachycardia/ventricular fibrillation + reversible causes (5H & 5T)

## Supporting information

S1 File Study data. (XLSX)
